# Supplementary material for: Characterization of Beverage Viscosity Based on the International Dysphagia Diet Standardisation Initiative and Its Correspondence to the Japanese Dysphagia Diet 2021
Source: Nutrients. 2025 Mar 17;17(6):1051. doi: 10.3390/nu17061051 (PMC11944815; doi:10.3390/nu17061051)
Supplement: Supplementary file 1 [file nutrients-17-01051-s001.zip › nutrients-3507339-supplementary.pdf]

Characterization of Beverage Viscosity Based on the International Dysphagia Diet Standardisation Initiative and its Correspondence to the Japanese Dysphagia Diet 2021

Table S1. Raw data of two measurements for each samples (n = 97)

Sample Number	Beverage	Viscosity	Thickener Amount	Syringe Residual	Funnel Residue	Fat content (g/100ml)	Sodium content (mg/100ml)	pH	Static time 1 min 5 s, 2 min 10 s
0	Mineral water	36	0.3	0.6	0	0	1	7	0
1	Mineral water	56	0.3	1.2	0	0	1	7	0
2	Mineral water	54	0.4	2.8	0.5	0	1	7	0
3	Mineral water	62	0.4	2.6	0	0	1	7	0
4	Mineral water	92	0.5	4	1	0	1	7	0
5	Mineral water	88	0.5	4.4	3.6	0	1	7	0
6	Mineral water	145	1	8.2	6	0	1	7	0
7	Mineral water	240	1	8.6	8	0	1	7	0
8	Mineral water	398	2	10	9.9	0	1	7	0
9	Mineral water	257	2	10	10	0	1	7	0
10	Green tea	11	0.3	0	0	0	0	6.5	0
11	Green tea	28	0.3	0	0	0	0	6.5	0
12	Green tea	53	0.4	0	0	0	0	6.5	0
13	Green tea	45	0.4	0	0	0	0	6.5	0

14	Green tea	79	0.5	0	0	0	0	6.5	0
15	Green tea	54	0.5	1.2	0	0	0	6.5	0
16	Green tea	245	1	6	2.2	0	0	6.5	0
17	Green tea	262	1	7.2	6	0	0	6.5	0
18	Green tea	443	1.6	9.9	8	0	0	6.5	0
19	Green tea	357	1.6	9.6	9.6	0	0	6.5	0
20	Orenge Juice (100%)	41	0.3	0	0	0.1	1	3.5	1
21	Orenge Juice (100%)	39	0.3	0	0	0.1	1	3.5	1
22	Orenge Juice (100%)	56	0.4	0	0	0.1	1	3.5	1
23	Orenge Juice (100%)	64	0.4	0	0	0.1	1	3.5	1
24	Orenge Juice (100%)	69	0.5	0	0	0.1	1	3.5	1
25	Orenge Juice (100%)	70	0.5	0	0	0.1	1	3.5	1
26	Orenge Juice (100%)	225	1	3.6	1.5	0.1	1	3.5	1
27	Orenge Juice (100%)	170	1	4.8	3	0.1	1	3.5	1

28	Orenge Juice (100%)	382	1.6	7	3.5	0.1	1	3.5	1
29	Orenge Juice (100%)	329	1.6	7.4	6	0.1	1	3.5	1
30	Milk	23	0.3	0	0	3.8	44	6.8	2
31	Milk	15	0.3	0.3	0.5	3.8	44	6.8	2
32	Milk	37	0.4	1	0.8	3.8	44	6.8	2
33	Milk	50	0.4	1	1.1	3.8	44	6.8	2
34	Milk	70	0.5	1.8	2	3.8	44	6.8	2
35	Milk	79	0.5	2.4	2	3.8	44	6.8	2
36	Milk	161	1	6.4	6	3.8	44	6.8	2
37	Milk	144	1	6.2	6.5	3.8	44	6.8	2
39	Milk	479	1.6	9	6	3.8	44	6.8	2
40	Coffee	45	0.3	0	0	0	2	5	0
41	Coffee	27	0.3	0	0	0	2	5	0
42	Coffee	54	0.4	0	0	0	2	5	0
43	Coffee	62	0.4	0	0	0	2	5	0
44	Coffee	52	0.5	0	0	0	2	5	0
45	Coffee	50	0.5	0	0	0	2	5	0
46	Coffee	154	1	6	2.3	0	2	5	0

47	Coffee	207	1	7.8	2.2	0	2	5	0
48	Coffee	401	1.8	9	6.4	0	2	5	0
49	Coffee	419	1.8	9.4	8.2	0	2	5	0
50	Black tea	48	0.5	0	0	0	1	5.5	0
51	Black tea	43	0.5	0	0	0	1	5.5	0
52	Black tea	46	0.6	1.2	0	0	1	5.5	0
53	Black tea	50	0.6	0.6	0	0	1	5.5	0
54	Black tea	80	0.7	3	0	0	1	5.5	0
55	Black tea	116	0.7	4	1.9	0	1	5.5	0
56	Black tea	241	1.2	7.4	5	0	1	5.5	0
57	Black tea	285	1.2	8	6.5	0	1	5.5	0
58	Black tea	463	1.6	9.8	8	0	1	5.5	0
59	Black tea	398	1.6	9.8	9.2	0	1	5.5	0
60	Oolong tea	29	0.5	0	0	0	3	6	0
61	Oolong tea	30	0.5	0	0	0	3	6	0
62	Oolong tea	40	0.6	0.1	0	0	3	6	0
63	Oolong tea	50	0.6	0.8	0	0	3	6	0
64	Oolong tea	127	0.7	1.6	0	0	3	6	0
65	Oolong tea	73	0.7	5.7	0.2	0	3	6	0
66	Oolong tea	227	1.2	7.6	4	0	3	6	0

67	Oolong tea	260	1.2	8.4	7	0	3	6	0
70	Sports drink	46	0.5	0	0.2	0	50	4.5	1
71	Sports drink	48	0.5	0	0	0	50	4.5	1
72	Sports drink	58	0.5	0	0.4	0	50	4.5	1
73	Sports drink	61	0.5	0.2	0.5	0	50	4.5	1
74	Sports drink	98	0.6	1	1	0	50	4.5	1
75	Sports drink	87	0.6	1	1	0	50	4.5	1
76	Sports drink	289	0.7	3.4	2.2	0	50	4.5	1
77	Sports drink	271	0.7	4.6	3.5	0	50	4.5	1
78	Sports drink	503	1.2	9.8	9.8	0	50	4.5	1
79	Sports drink	459	1.2	9.8	9.8	0	50	4.5	1
82	Tomato juice	126	0.6	9.8	4.8	0	25	4.2	1
83	Tomato juice	122	0.6	9.8	4.4	0	25	4.2	1
84	Tomato juice	159	0.7	5.4	4.4	0	25	4.2	1
85	Tomato juice	150	0.7	5.2	4.8	0	25	4.2	1
86	Tomato juice	199	1.2	6.6	4.6	0	25	4.2	1
87	Tomato juice	197	1.2	7	6	0	25	4.2	1
88	Tomato juice	435	2	8.8	9	0	25	4.2	1
89	Tomato juice	414	2	9.2	9	0	25	4.2	1
90	Lactic acid	131	0.5	0	0	0.1	15	3.6	2

	beverage								
91	Lactic acid beverage	166	0.5	0	0	0.1	15	3.6	2
92	Lactic acid beverage	121	0.6	0	0	0.1	15	3.6	2
93	Lactic acid beverage	185	0.6	0	0	0.1	15	3.6	2
94	Lactic acid beverage	175	0.7	0	0	0.1	15	3.6	2
95	Lactic acid beverage	166	0.7	0	0	0.1	15	3.6	2
96	Lactic acid beverage	274	1.2	2	2	0.1	15	3.6	2
97	Lactic acid beverage	267	1.2	3	3	0.1	15	3.6	2
98	Lactic acid beverage	475	1.8	10	10	0.1	15	3.6	2
99	Lactic acid beverage	451	1.8	10	10	0.1	15	3.6	2
